# FLOW800−guided microsurgical resection of a brainstem hemangioblastoma with AVM−like features: a case report and technical note

**DOI:** 10.3389/fonc.2026.1856923

**Published:** 2026-06-03

**Authors:** Ying Yang, Chunfeng Pan, Chuan He, Zhaobin Chen, Zhaoyi Quan, Gang Cao

**Affiliations:** 1 Department of Neurosurgery, Zhuhai Hospital of Integrated Traditional Chinese and Western Medicine(Zhuhai Hospital Affiliated to Faculty of Chinese Medicine, Macau University of Science and Technology), Zhuhai, China; 2 Zhuhai Integrated Traditional Chinese and Western Medicine Hospital Gongbei Community Health Service Center, Zhuhai, China; 3 Department of Neurosurgery, Lufeng Second People’s Hospital (Lufeng Jiazi People’s Hospital), Shanwei, China; 4 Department of Neurosurgery, Heping County People’s Hospital, Heping, China

**Keywords:** brainstem hemangioblastoma, case report, FLOW800, fluorescence angiography, microsurgery

## Abstract

**Background:**

Brainstem hemangioblastomas (HBs) are rare, highly vascularized benign tumors that pose significant surgical challenges due to their deep location, proximity to critical neural structures, and arteriovenous malformation (AVM)-like angioarchitecture. Preoperative embolization carries a high risk of edema or hemorrhage, and piecemeal resection is often impossible. The intraoperative use of FLOW800 color-coded fluorescence angiography may improve real-time hemodynamic assessment, but its application in brainstem HB surgery is rarely reported.

**Case presentation:**

A 39-year-old female presented with headache, dizziness, and ataxia. Imaging revealed a brainstem HB involving the fourth ventricle and cerebellar vermis, with obstructive hydrocephalus and syringomyelia. The patient underwent a suboccipital median approach. Intraoperatively, FLOW800 under a Zeiss microscope was used to differentiate feeding arteries from draining veins. Following FLOW800 guidance, the tumor was dissected circumferentially with en bloc resection, paying extreme gentleness to the brainstem interface, especially at the lower pole adherent to the medulla oblongata. Gross-total resection (2.3 cm × 3.0 cm × 2.4 cm) was achieved with minimal blood loss. The patient recovered well, was extubated 4 hours postoperatively, and had no new cranial nerve or respiratory deficits. Histopathology and immunohistochemistry (inhibin-α+/CD10–/GFAP–/EMA–) confirmed HB and excluded mimics.

**Conclusion:**

For brainstem HBs with AVM-like hypervascularity, FLOW800-guided en bloc peripheral dissection combined with ultra-gentle manipulation of the tumor-brainstem interface allows safe gross-total resection without preoperative embolization. This case supports the routine intraoperative use of FLOW800 in selected brainstem HBs.

## Introduction

Hemangioblastomas (HBs) are highly vascularized, slow-growing, and relatively rare benign tumors composed of stromal and capillary cells. They account for approximately 2% of intracranial tumors and most commonly arise in the cerebellum. However, when the brainstem is involved, treatment often becomes particularly challenging. The present case illustrates this clinical dilemma: the lesion involved the brainstem and compressed the pons and medulla oblongata, resulting in a restricted surgical corridor, yet the patient presented with only mild clinical symptoms on admission. Intraoperatively, the tumor was highly vascular, precluding piecemeal resection; instead, a rapid en bloc resection had to be achieved by gradually dissecting the periphery. The intraoperative use of the FLOW800 mode on a Zeiss microscope, which generates color-coded delay maps of blood flow in the exposed surgical field, allowed for real-time assessment and analysis of regional hemodynamics, rapid identification of feeding arteries, and determination of main draining veins, thereby providing crucial support for the surgical procedure.

## Case presentation

A 39-year-old female presented with a one-week history of headache and dizziness. On admission, she was alert and oriented with fluent speech. Her pupils were equal and round (diameter 3 mm) with prompt light reflexes. The remainder of the cranial nerve examination was unremarkable. Muscle strength and tone in all four limbs were normal, but ataxia was present. Bilateral pathological reflexes and meningeal signs were negative.

Head CT revealed an occupying lesion involving the brainstem, fourth ventricle, and cerebellar vermis, accompanied by obstructive hydrocephalus. Further head and neck CT angiography (CTA) showed the lesion to be hypervascular (**Figure 1**). Multi-parametric contrast-enhanced MRI demonstrated a heterogeneous signal: iso-intense on T1-weighted imaging with multiple small vacuolated hypo-intensities; slightly hyper-intense on T2-weighted imaging with multiple small vacuolated hyper-intensities; hyper-intense on FLAIR; hypo-intense on DWI. Post-contrast imaging showed rapid and marked enhancement, with flow voids around the lesion. The mass involved the brainstem and displaced the cerebellar vermis and tonsils upward. Correspondingly, ventricular dilatation was noted, and the upper spinal cord exhibited patchy hypo-intensity on T1-weighted and hyper-intensity on T2-weighted images (**Figure 1**). Based on these findings, a diagnosis of brainstem hemangioblastoma with syringomyelia and obstructive hydrocephalus was made. Whole-body CT of the chest and abdomen ruled out lesions in the kidneys or pancreas, supporting a sporadic form of hemangioblastoma.

**Figure 1:**
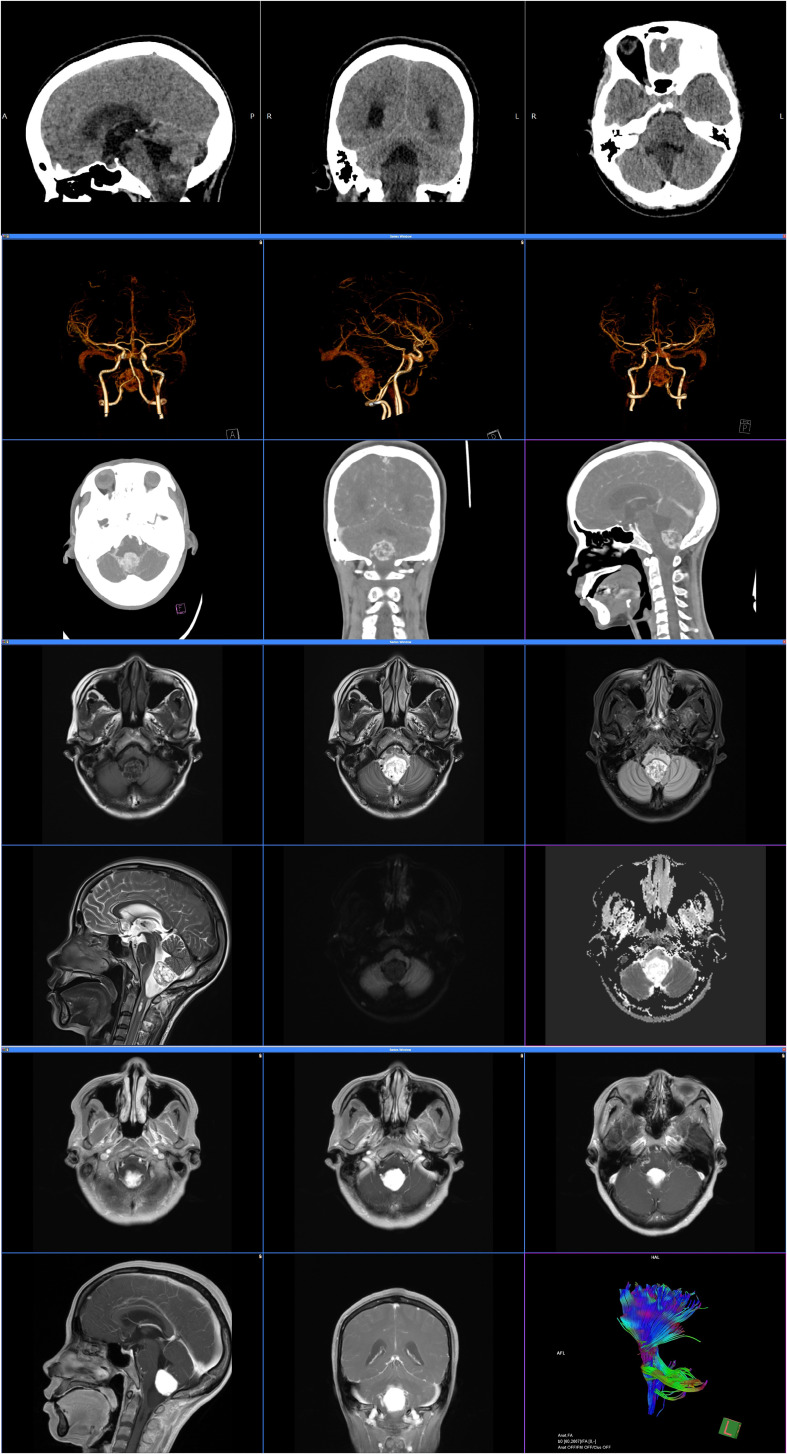
Admission CT scan showing an occupying lesion in the medulla oblongata and fourth ventricle, with obstructive hydrocephalus. Preoperative CTA showing the lesion to be hypervascular. Preoperative MRI: T1-weighted image showing iso-intensity with multiple small vacuolated hypo-intensities; T2-weighted image showing slightly hyper-intensity; DWI showing hypo-intensity. Preoperative contrast-enhanced MRI showing rapid and marked enhancement of the lesion.

The patient underwent a suboccipital median approach for resection of the brainstem hemangioblastoma. Intraoperatively, cerebrospinal fluid (CSF) was drained via puncture of the right occipital horn of the lateral ventricle to reduce intracranial pressure. The posterior arch of C1 was drilled away to achieve adequate decompression, and the dura was opened. The tumor appeared reddish, highly vascular, with distinctly arterialized peripheral veins. The lower pole of the tumor contained a cystic component consistent with the MRI findings, and the surrounding space was severely compressed by the mass. Using FLOW800 fluorescence angiography under a Zeiss microscope, feeding arteries and main draining veins were identified (**Figure 2**). The tumor boundary was selectively and gradually dissected, particularly at the lower pole where the tumor adhered to the medulla oblongata. Finally, gross total resection was achieved with minimal blood loss. The tumor measured 2.3 cm × 3.0 cm × 2.4 cm.

**Figure 2:**
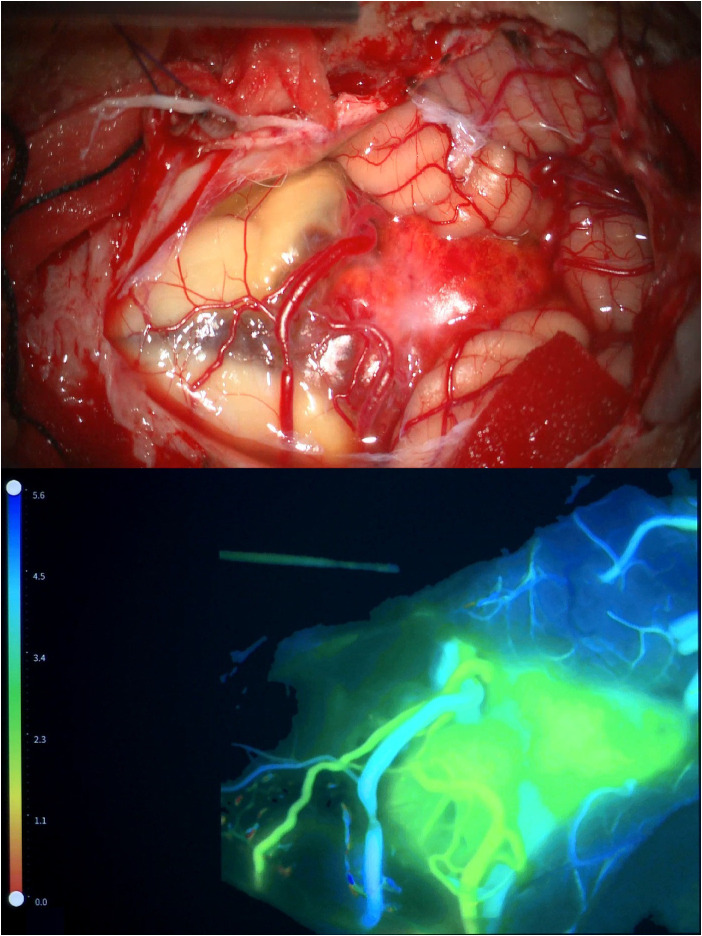
Intraoperative FLOW800 imaging demonstrating distinct temporal delay differences among various vessels. and corresponding microscopic view.

Postoperatively, the patient’s respiratory function recovered well, and she was successfully extubated 4 hours after surgery. No dysphagia, hoarseness, or choking on liquids occurred, and no cranial nerve deficits were observed. **Figure 3** shows the patient’s postoperative head CT and contrast-enhanced MRI. The resected specimen was sent for histopathological examination, which confirmed hemangioblastoma. Immunohistochemistry revealed: Immunohistochemically, the stromal cells were positive for inhibin-α and vimentin, while negative for CD10, GFAP, and EMA. The vascular endothelial cells showed diffuse positivity for CD31 and CD34. Additional positive markers included D2–40 and CD56(**Figure 4**). This profile (inhibin-α+/CD10–/GFAP–/EMA–) is diagnostic of hemangioblastoma and effectively excludes its main mimics, particularly metastatic clear cell renal cell carcinoma and glioma.

**Figure 3:**
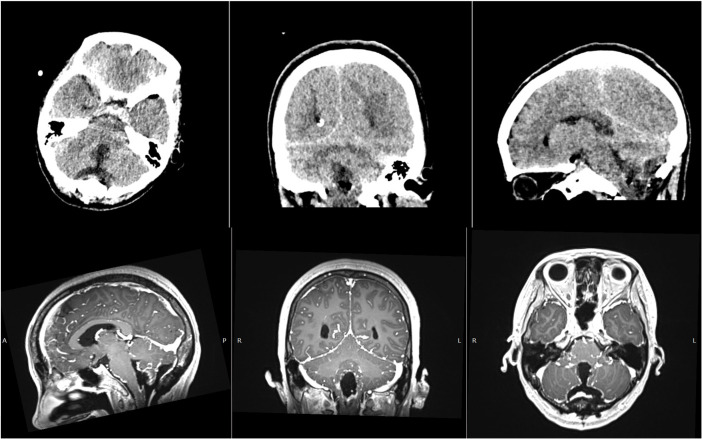
Postoperative CT scan showing gross total resection of the lesion and improvement of hydrocephalus. Postoperative contrast-enhanced MRI showing complete resection of the mass with good preservation of the surrounding brain tissue.

**Figure 4:**
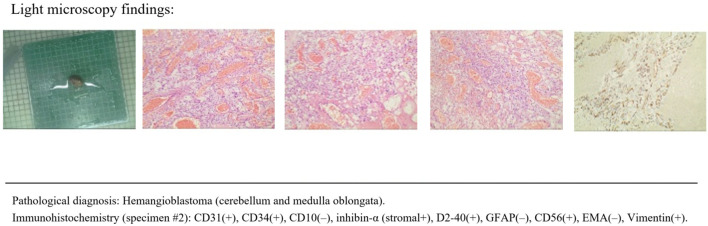
Histopathology (H&E and inhibin α immunohistochemistry).

## Discussion

This case illustrates a sporadic brainstem hemangioblastoma (HB) that presented with only mild clinical symptoms despite significant mass effect and obstructive hydrocephalus. The tumor’s location – involving the brainstem, fourth ventricle, and cerebellar vermis – posed substantial surgical challenges (1). Here we discuss three key aspects that determined the success of the resection: the inherent arteriovenous malformation (AVM)-like features of brainstem HBs (2), the intraoperative use of FLOW800 fluorescence angiography (3), and the delicate microsurgical techniques required for safe dissection along the brainstem surface.

First, brainstem HBs share several angiographic and hemodynamic characteristics with AVMs. They are typically supplied by multiple enlarged feeding arteries, exhibit early venous drainage, and contain a dense capillary network interwoven with stromal cells. In our patient, intraoperative findings confirmed a highly vascular, reddish tumor with distinctly arterialized peripheral veins – features that closely mimic an AVM. Such vascular architecture precludes piecemeal resection, as any attempt to debulk the tumor internally would result in catastrophic bleeding and obscure the surgical field (4–6). Consequently, the only safe strategy is to achieve en bloc resection by meticulously dissecting the tumor periphery. Preoperative endovascular embolization has been proposed for some hypervascular HBs to reduce intraoperative bleeding (7). However, in the brainstem, the risks of post-embolization edema, intratumoral haemorrhage, or rupture of feeding vessels may outweigh the benefits. In selected larger or recurrent tumours, embolization could be considered, but for this solitary, moderately sized brainstem HB, we opted for direct microsurgical resection without prior embolisation to avoid potential catastrophic complications. This approach demands exceptional microsurgical skill, including the ability to identify and temporarily control feeding arteries under high magnification while preserving the surrounding venous drainage until the final stage of resection (8).

Second, the FLOW800 color-coded fluorescence angiography integrated into the Zeiss microscope proved invaluable for real-time hemodynamic assessment. Unlike conventional indocyanine green (ICG) angiography, which provides only binary (on/off) information, FLOW800 generates quantitative delay maps that display the temporal dynamics of blood flow (3, 9). In our case, FLOW800 enabled rapid differentiation between feeding arteries (early filling) and draining veins (delayed filling) even before the tumor surface was fully exposed. This information guided the sequence of vessel coagulation: we first interrupted the arterial supply at the tumor-brainstem interface, particularly at the lower pole where the tumor adhered to the medulla oblongata, while leaving the main draining veins intact to prevent venous congestion. Only after complete circumferential dissection and devascularization were the draining veins divided. Without FLOW800, the surgeon would have to rely solely on visual color cues and pulsation, which can be misleading in a crowded, bloody field. Several previous reports have described the utility of FLOW800 in cerebral AVM and aneurysm surgery (3, 10, 11), but its application in brainstem HB resection remains less documented. Our experience confirms that FLOW800 significantly enhances safety by reducing the risk of premature venous sacrifice and inadvertent injury to en passage vessels that supply the brainstem. Although FLOW800 has been increasingly used in cerebral AVM and aneurysm surgery to map flow dynamics and guide vessel sacrifice, its application in brainstem HBs is distinct because the tumour’s AVM-like angioarchitecture coexists with intimately adherent brainstem parenchyma. Unlike in an AVM nidus, the feeding arteries in brainstem HBs are often short and directly enter the tumour capsule, while draining veins are arterialized and fragile. FLOW800 not only differentiates feeders from drainers but also helps avoid inadvertent injury to en passage vessels that supply the medulla – a risk less frequently encountered in supratentorial AVM surgery. We acknowledge that standard ICG videoangiography (without FLOW800) can also differentiate arteries from veins based on the chronological order of fluorescence; however, FLOW800’s colour-coded delay maps offer a more intuitive and quantitative display, which is particularly helpful in deep and narrow surgical corridors such as the brainstem. Nevertheless, this advantage is based on a single case and requires further comparative evaluation.

Third, the separation sequence and the handling of the brainstem surface require extreme gentleness. In this patient, the tumor had displaced the cerebellar vermis and tonsils upward, and its lower pole was firmly adherent to the medulla oblongata (8). The standard sequence for HB resection is: (1) identify the arterial feeders using FLOW800, (2) coagulate and divide the feeders along the tumor capsule, (3) develop a dissection plane circumferentially, (4) gently retract the tumor away from the brainstem, and (5) finally divide the draining veins. During step 4, we deliberately avoided applying any suction or traction directly on the brainstem side. Instead, the tumor was gently flipped outward after the inferior pole was fully mobilized, and any residual adhesion was cut with microscissors under continuous irrigation. Excessive manipulation of the medulla can provoke bradycardia, hypotension, or postoperative respiratory compromise. The fact that our patient was extubated successfully 4 hours after surgery without swallowing difficulties or vocal cord palsy reflects the benefit of this atraumatic technique. We also emphasize that the cystic component at the lower pole, as seen on preoperative MRI, should not be punctured prematurely, because decompressing the cyst may collapse the dissection plane and increase the risk of tearing small feeding vessels. The cyst wall itself is part of the tumor and should be resected en bloc with the solid component.

### Histopathological correlation

The resected specimen showed classical biphasic architecture of hemangioblastoma. Haematoxylin and eosin staining revealed numerous thin-walled capillary-sized vessels admixed with clusters of large polygonal stromal cells. These stromal cells had well-defined borders, clear to eosinophilic cytoplasm, and round to oval nuclei without significant atypia (**Figure 4**). Immunohistochemistry demonstrated strong cytoplasmic and nuclear staining for inhibin-α in the stromal cells, while the endothelial cells were positive for CD31 and CD34. The complete absence of CD10, GFAP and EMA expression effectively ruled out metastatic clear cell renal cell carcinoma, glioma and meningioma. This immunoprofile is diagnostic of central nervous system HB and supports the sporadic nature of the lesion.

In summary, this case demonstrates that for brainstem hemangioblastomas with AVM-like hypervascularity, a strategy combining FLOW800-guided real-time angiography, en bloc peripheral dissection, and ultra-gentle manipulation of the brainstem interface can achieve gross total resection with minimal morbidity (8, 12). A limitation of this report is its single-case nature; the generalizability of FLOW800 assistance in brainstem HB surgery requires further validation through larger series or comparative studies. Nevertheless, for neurosurgeons facing similarly challenging brainstem HBs, we recommend routine integration of FLOW800 into the operative armamentarium, along with strict adherence to the principles of vascular control and brainstem preservation.

## Conclusion

This case of a sporadic brainstem hemangioblastoma with syringomyelia and obstructive hydrocephalus highlights that despite its benign histology, the tumour’s hypervascular, AVM-like angioarchitecture poses substantial surgical risks when located in the brainstem. Gross-total resection can be safely achieved without preoperative embolization by adhering to a strategy of en bloc peripheral dissection under the guidance of FLOW800 fluorescence angiography. The FLOW800 mode provides real-time, colour-coded haemodynamic maps that enable rapid identification of feeding arteries and main draining veins, thereby reducing the risk of premature venous sacrifice and inadvertent injury to brainstem perforators. Equally important is the ultra-gentle handling of the tumour-brainstem interface, particularly at the lower pole where adherence to the medulla oblongata is most firm. With this combined approach, our patient recovered rapidly without new neurological deficits. Given the single-case nature of this report, we suggest that for similarly challenging brainstem hemangioblastomas, neurosurgeons may consider intraoperative FLOW800 as a valuable adjunct to microsurgical technique, while recognizing that its added value needs confirmation through larger series or comparative studies.

## Data Availability

The original contributions presented in the study are included in the article/**Supplementary Material**. Further inquiries can be directed to the corresponding author.
